# Oral immunization with a novel attenuated *Salmonella* Typhimurium encoding influenza HA, M2e and NA antigens protects chickens against H7N9 infection

**DOI:** 10.1186/s13567-018-0509-y

**Published:** 2018-02-01

**Authors:** Je Hyoung Kim, Irshad Ahmed Hajam, John Hwa Lee

**Affiliations:** College of Veterinary Medicine, Chonbuk National University, Iksan, 54596 Republic of Korea

## Abstract

Attenuated *Salmonella* strains constitute a promising technology for the development of efficient protein-based influenza vaccines. H7N9, a low pathogenic avian influenza (LPAI) virus, is a major public health concern and currently there are no effective vaccines against this subtype. Herein, we constructed a novel attenuated *Salmonella* Typhimurium strain for the delivery and expression of H7N9 hemagglutinin (HA), neuraminidase (NA) or the conserved extracellular domain of the matrix protein 2 (M2e). We demonstrated that the constructed *Salmonella* strains exhibited efficient HA, NA and M2e expressions, respectively, and the constructs were safe and immunogenic in chickens. Our results showed that chickens immunized once orally with *Salmonella* (Sal) mutants encoding HA (Sal-HA), M2e (Sal-M2e) or NA (Sal-NA), administered either alone or in combination, induced both antigen-specific humoral and cell mediated immune (CMI) responses, and protected chickens against the lethal H7N9 challenge. However, chickens immunized with Sal-HA+Sal-M2e+Sal-NA vaccine constructs exhibited efficient mucosal and CMI responses compared to the chickens that received only Sal-HA, Sal-M2e or Sal-M2e+Sal-NA vaccine. Further, chickens immunized with Sal-HA+Sal-M2e+Sal-NA constructs cleared H7N9 infection at a faster rate compared to the chickens that were vaccinated with Sal-HA, Sal-M2e or Sal-M2e+Sal-NA, as indicated by the reduced viral shedding in cloacal swabs of the immunized chickens. We conclude that this vaccination strategy, based on HA, M2e and NA, stimulated efficient induction of immune protection against the lethal H7N9 LPAI virus and, therefore, further studies are warranted to develop this approach as a potential prophylaxis against LPAI viruses affecting poultry birds.

**Electronic supplementary material:**

The online version of this article (10.1186/s13567-018-0509-y) contains supplementary material, which is available to authorized users.

## Introduction

Avian influenza viruses, classified as highly pathogenic avian influenza (HPAI) or low-pathogenicity avian influenza (LPAI), cause huge economical losses to the poultry industry annually [1, 2]. Outbreaks of HPAI and LPAI viruses belonging to H7 subtype in chickens have been reported in the past [3–5], and infections caused by H7N9 LPAI virus have not only infected poultry birds but also humans as well, with limited person to person transmission [6, 7]. The World Health Organization (WHO) has identified H7N9 virus as an unusually dangerous virus for humans, and infection caused by H7N9 virus is a major public health concern as it is unlikely that there will be pre-existing immunity against this subtype in the human population [8]. Humans infected with H7N9 virus mostly result in severe respiratory illness, with a mortality of roughly 30% [9]. The H7N9 virus possess characteristic features related to the human adaptation, for instance mutations in the HA and PB2 proteins, which facilitate the virus binding to and replicating in the respiratory tract [7, 10]. Most of the H7N9 vaccines showed low immunogenicity and induction of non-protective hemagglutination-inhibiting antibody titers [11, 12]. Thus, potent and effective vaccines must be available to prevent the occurrence of H7N9 infection in humans. Recently, a complete protection against the lethal challenge of novel H7N9 virus with heterologous inactivated H7 vaccine was observed in mice [13]. Although effective in controlling the H7N9 infection, these inactivated influenza vaccines require a large supply of specific-pathogen free (SPF) embryonated eggs and a long timeline that could be threatened during an influenza pandemic affecting both animal and human population [14]. Thus, effective vaccination strategies should be in place which not only induce protective immune responses but concurrently allows easier manipulation and faster production of vaccines, and can provide broader spectrum of protection against the heterologous strains of influenza viruses [14]. Earlier studies have reported that live attenuated *Salmonella*-based vaccines carrying various heterologous bacterial or viral antigens elicit efficient protective mucosal and cell mediated immune (CMI) responses [15]. *Salmonella*-based vaccination strategy is highly economical and allows for a quick response to novel influenza viruses, as it eliminates the need for specific pathogen free (SPF) embryonated eggs required for the production of conventional influenza vaccines. Moreover, this strategy allows the differentiation of vaccinated from infected animals as antigenically and immunologically relevant antigens are used to construct the vaccine strain.

Previously, we have constructed an O antigen intact *Salmonella*-hemagglutinin (HA) consensus (H7N3, H7N7 and H7N9 sequence) based vaccine that conferred significant protection against the lethal heterologous H7N1 infection in chicken model [15]. The HA is the most abundant integral viral envelope protein and is the major target for generating protective immunity; however, HA is continuously subjected to antigenic variations either through antigenic drift (point mutations) or rapidly through reassortment with another divergent virus (antigenic shift). Consequently, the immunity generated against one vaccine strain is only protective against another strain that shares antigenically related proteins. Similar to HA, the matrix protein 2 (M2) is an integral transmembrane protein of influenza A viruses [16]. The ectodomain of M2 (M2e), 23 amino acid residues, has been remarkably conserved in all human influenza A strains, and animal experiments have demonstrated that M2e-specific antibodies can provide cross protective immunity against infections caused by different types of influenza A subtypes [16]. Recently, Schotsaert et al. has shown that neuraminidase (NA) and M2e-based immunization strategies can induce long-lasting cross-protective immunity against influenza A viruses [17]. In accordance with this notion, in this study, we constructed O antigen deficient attenuated auxotrophic mutant of *Salmonella* Typhimurium expressing and secreting HA, M2 ectodomain (M2e) or NA of H7N9 virus, and evaluated the efficacy of these *Salmonella*-based influenza vaccines in chicken model.

Our results show that chickens immunized once orally with *Salmonella* mutants encoding HA (Sal-HA), M2e (Sal-M2e) or NA (Sal-NA), administered either alone or in combination, induced both humoral and CMI responses, and protected the chickens against the lethal H7N9 challenge. Our results further demonstrate that the chickens immunized with a co-mix of *Salmonella* mutants encoding HA, M2e or NA proteins showed higher protective immunity than the chickens vaccinated with Sal-HA, Sal-M2e or Sal-M2e+Sal-NA based vaccine.

## Materials and methods

### Virus and cell line

The tissue culture infective dose (TCID_50_) of H7N9 influenza virus, cultivated in the allantoic cavity of SPF embryonated eggs, was calculated in Madin Darby Canine Kidney (MDCK) cells as described previously [15].

### Construction of O antigen deficient attenuated auxotrophic *Salmonella* Typhimurium mutants expressing HA, M2e tetramer or NA antigens

The HA consensus sequence derived from H7N3, H7N7 and H7N9 viruses was constructed and built into pMMP65 expression vector as described elsewhere [15]. The four tandem repeats of M2e sequence (MSLLTEVETPTRNGWECKCSDSSD) was constructed and built into pMMP65 vector as described elsewhere [18]. The NA consensus sequence was deduced from 605 sequences of H7N9 virus strains based on the data available in the GenBank (Additional file 1) and optimized for efficient gene expression in *Salmonella*, then synthesized (Bionee, Korea) and built into the pMMP65 vector as described earlier [15]. The recombinant plasmids pMMP65-HA, pMMP65-4M2e or pMMP65-NA were subsequently transformed into an O antigen deficient auxotrophic mutant of *S*. Typhimurium, strain JOL1800, and the resultant clones were designated as JOL2030 (Sal-HA), JOL1913 (Sal-M2e) and JOL2052 (Sal-NA), respectively. The O antigen deficient *S*. Typhimurium mutant was constructed by the deletion of the *lon*, *cpxR*, *asd* and *wbaP* genes as described elsewhere [18], and used as the delivery system for the influenza antigens. The protein expressions in *Salmonella* system were confirmed by the Western blot analysis as previously described [15, 18]. The bacterial strains and plasmids used in this study are listed in Table 1.

**Table 1:** **Bacterial strains and plasmids used in this study**

	Description	References
Strains/plasmids		
JOL1800	*∆lon*, *∆cpxR*, *∆asd*, *wbaP* mutant of *S.* Typhimurium	[18]
JOL1837	JOL1800 with pMMP65	[18]
JOL1913	JOL1800 with pMMP65-4M2e	[18]
JOL2030	JOL1800 with pMMP65-HA1 plasmid	This study
JOL2052	JOl1800 with pMMP65-NA	This study
Plasmids		
pET28(+)	IPTG-inducible, T7 expression vector, C-terminal 6× His tag, Kan^R^	Novagen, USA
pMMP65	*asd+*, pBR*ori*, lactamase signal sequence based periplasmic secretion plasmid, 6 His tag, high copy number plasmid	[38]

### Immunization and challenge studies of chickens

All animal experimentation work was approved by the Chonbuk National University Animal Ethics Committee (CBU 2014-1-0038) and the chicken experiments were carried out according to the guidelines of the Korean Council on Animal Care. One-day-old female layer chickens (Corporation of Join hatchery, Republic of Korea) were maintained under standard conditions and provided antibiotic-free food and water ad libitum. Two weeks later, the chickens were randomly divided into five groups (*n* = 12) and immunized orally with *Salmonella*-based influenza vaccines. Group 1 (Sal-vector) received JOL1837 (JOL1800 carrying an empty pMMP65 vector), group 2 (Sal-HA) received JOL2030, group 3 (Sal-M2e) received JOL1913, group 4 (Sal-M2e-NA) received a co-mix of JOL1913 and JOL2052, and group 5 received a co-mix of JOL2030, JOL1913 and JOL2052. The 10^9^ colony forming units (CFU) of each bacterial strain were used for inoculation in this study. Serum and intestinal wash samples were collected on the day of vaccination (pre-vaccination) and weekly thereafter to assess the HA, M2e and NA specific systemic IgG and mucosal IgA humoral responses.

Four weeks post-vaccination, all the immunized and the control chickens were intranasally challenged with 1.6 × 10^4^ TCID_50_ of H7N9 virus and the cloacal swabs were collected through day 1–day 6 post-challenge to determine the viral load by qRT-PCR assay as described previously [15]. For histological analyses, three chickens in each group were sacrificed on day 4 post-challenge. Lung and intestinal tissues were aseptically collected and fixed with 10% formalin, embedded in paraffin and cut into 5 μm thin sections. These sections were subsequently stained with hematoxylin and eosin (H&E) for microscopic examination.

### Hemagglutination inhibition assay and virus neutralization assay

Hemagglutination inhibition (HI) assay was performed to measure the HI antibody titers in the sera of vaccinated and control chickens as described earlier [15, 18].

Neutralizing activity of the immunized sera against H7N9 virus was measured by micro-neutralization assay as per the recommendations of the WHO with slight modifications. Briefly, complement inactivated sera (50 μL) were diluted twofold in modified eagle’s medium (MEM) across column of a 96-well tissue culture plate (SPL Life Sciences, Korea). Then, a 50 μL of MEM containing 100TCID_50_ H7N9 virus was added to each well and the mixture incubated at 37 °C for 1 h. Thereafter, a 50 μL suspension of MDCK cells (2 × 10^4^) was added to each well and the plate was further incubated at 37 °C for 24 h in a humidified chamber containing 5% CO_2_. Virus, serum and cell controls were included in each test. After 24 h of incubation, the plates were washed with sterile PBS and the virus neutralizing antibody titers were calculated using a colorimetric MTT based method as described in Bantia et al. [19]. The serum neutralizing antibody end point titers were determined utilizing the equation below:

$${\text{X}}\; = \;{{\left( {{\text{mean}}\;{\text{OD}}\;{\text{of}}\;{\text{VC}}\;{\text{wells}}\; - \;{\text{mean}}\;{\text{OD}}\;{\text{of}}\;{\text{CC}}\;{\text{wells}}} \right)} \mathord{\left/ {\vphantom {{\left( {{\text{mean}}\;{\text{OD}}\;{\text{of}}\;{\text{VC}}\;{\text{wells}}\; - \;{\text{mean}}\;{\text{OD}}\;{\text{of}}\;{\text{CC}}\;{\text{wells}}} \right)} 2}} \right. \kern-0pt} 2}\; + \;\left( {{\text{mean}}\;{\text{OD}}\;{\text{of}}\;{\text{CC}}\;{\text{wells}}} \right)$$

where X = 50% of specific signal (i.e. 50% infected cells), VC is virus control and CC is cell control.

Immunized sera showing values below X values are positive for neutralization activity and the serum neutralization titers are calculated as log10 of the reciprocal of the last serum dilution that neutralized the H7N9 virus activity by 50%.

### Systemic IgG and mucosal IgA specific antibody responses

The systemic HA, M2e and NA specific IgG and mucosal IgA antibody levels in sera and in intestinal wash samples, respectively, were analysed by an indirect ELISA as described previously [15, 20].

### Lymphocyte proliferation assay

The cell mediated immunity elicited by vaccination was evaluated by a lymphocyte proliferation test as described previously [15]. Two weeks post-immunization, in vitro proliferation of vaccinated peripheral blood mononuclear cells (PBMCs) in response to the inactivated H7N9 recall antigen was measured by a MTT [3-(4,5-dimethylthiazol-2-yl)-2,5-diphenyltetrazolium bromide] based assay as described in Hyoung et al. [15].

### qRT-PCR assay

PBMCs restimulated with inactivated H7N9 virus were harvested after 24 h and total RNA isolated from stimulated cells was analysed for IFN-γ, IL-4 and IL-17 gene expressions by qRT-PCR assay as described previously [15].

### Statistical analysis

All the obtained data was analysed using GraphPad prism 6.00 program (San Diego, CA, USA). One way ANOVA with Tukey’s multiple comparison test was used to compare the data between different groups. *p* < 0.05 were considered statistically significant.

## Results

### Construction of a HA, M2e or NA based influenza vaccine delivered by an O antigen deficient attenuated auxotrophic mutant of *Salmonella* Typhimurium

The construction of *Salmonella*-HA and M2e-based influenza vaccines is described elsewhere [15, 18]. To construct the *Salmonella*-NA based influenza vaccine, the codon optimized NA gene sequence was cloned in frame into pMMP65 expression vector as described earlier [15]. The presence of NA gene in pMMP65 vector was confirmed by digestion of recombinant clones with *Eco*R1 and *Hind*III to release a fragment of 1425 bp size. The recombinant plasmid, pMMP65-NA, was transformed into JOL1800 strain for expression of NA protein as described previously [18], and the resultant clone was designated as JOL2052. Western blot analysis with a polyclonal NA antibody (catalog#, NBP2-41279, Novus Biologicals USA) showed an immunoreactive band corresponding to the ∼58 kDa, the expected size of our protein of interest, and thus confirmed the NA expression (Additional file 2).

### Immunization with HA, M2e and NA antigens delivered by *Salmonella* system induces protective antibody responses

**Figure 1:**
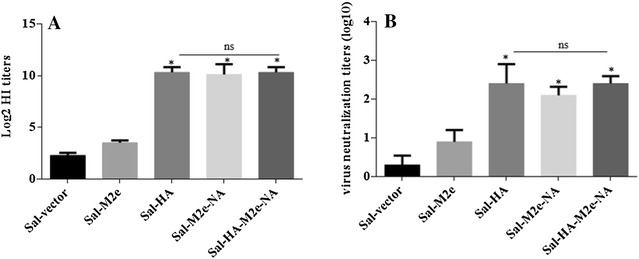
**HI and virus neutralizing antibody titers in chickens after immunization with** ***Salmonella*-based influenza vaccines.** Chickens (*N* = 12) were immunized with Sal-vector, Sal-HA, Sal-M2e, Sal-M2e-NA or Sal-HA-M2e-NA vaccine, and serum samples were analysed for HI (**A**) and VN titers (**B**) after 28 days post-vaccination. Each data points represent mean ± SD of five chickens per group. *p* < 0.05. ns: non-significant.

HI and virus neutralization (VN) tests reflect the functional antibody levels that confer protective immunity against the influenza infections [14]. To investigate the ability of *Salmonella* system delivering influenza antigens to induce HI and VN antibody titers, blood was drawn from vaccinated and naïve chickens on 28^th^ day post-immunization. Our results demonstrated that efficient HI and neutralizing antibody responses were induced in immunized chickens with respect to the control group (Figure 1). As shown in Figure 1A, the HI titers were significantly (*p* < 0.05) higher in vaccinated chickens as compared to the control group. However, no statistical significant difference was observed when HI titers were compared among groups immunized with *Salmonella*-HA, *Salmonella*-M2e-NA or *Salmonella*-HA-M2e-NA based vaccine.

We also analysed VN titers among immunized chickens by serum microneutralization assay. Our results showed that VN titers were significantly (*p* < 0.05) higher in immunized chickens as compared to the control group (Figure 1B). Among immunized groups, chickens vaccinated with *Salmonella*-M2e based vaccine showed significantly (*p* < 0.05) lower VN titers compared to the groups immunized with *Salmonella*-*HA*, *Salmonella*-M2e-NA or *Salmonella*-HA-M2e-NA based vaccine. However, no statistical significant difference was observed when VNT were compared among Sal-HA, Sal-M2e-NA and Sal-HA-M2e-NA vaccinated groups. These findings suggest that *Salmonella*-based influenza vaccines have potential to elicit significant neutralizing antibody responses which reflect the level of protective immunity against influenza challenges.

### Immunization with HA, M2e and NA antigens delivered by *Salmonella* system elicits efficient systemic and mucosal antibody responses

**Figure 2:**
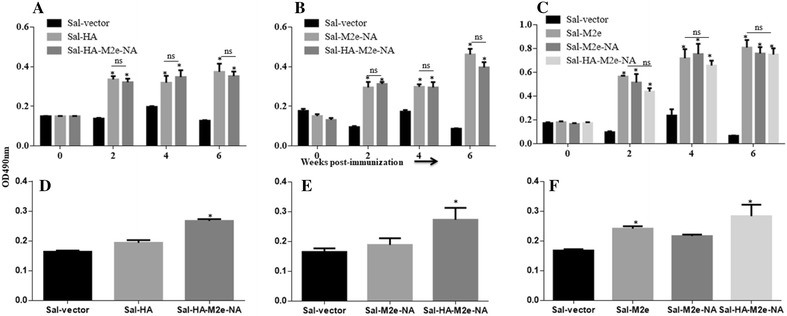
***Salmonella*-based influenza vaccines elicitate systemic IgG and mucosal IgA responses.** Chickens (*N* = 12) were immunized with Sal-vector, Sal-HA, Sal-M2e, Sal-M2e-NA or Sal-HA-M2e-NA vaccine, and serum and intestinal wash samples were analysed for IgG and sIgA levels, respectively, by indirect ELISA. Serum IgG responses were measured at different time points post-vaccination while sIgA levels were measured at 28^th^ day post-immunization. **A** HA-specific serum IgG responses, **B** NA-specific serum IgG responses, **C** M2e-specific serum IgG responses, **D** HA-specific sIgA responses, **E** NA-specific sIgA responses, and **F** M2e-specific sIgA responses. Each data points represent mean ± SD of five chickens per group. *p < 0.05. ns: non-significant.

To investigate the effect of immunization on systemic and mucosal antibody responses, indirect ELISA for IgG and IgA was performed on weekly collected post-vaccination sera and intestinal wash samples, respectively. The HA, M2e and NA specific IgG and IgA responses were induced following vaccination with *Salmonella*-based influenza vaccines. The kinetics of IgG and IgA antibodies after vaccination are shown in Figure 2.

Chickens vaccinated with either *Salmonella*-HA or *Salmonella*-HA-M2e-NA based vaccine showed significantly (*P* < 0.05) higher HA-specific systemic IgG responses as compared to the control chicken group (Figure 2A). The IgG levels were detected at 14^th^ day post-immunization, which maintained till 6^th^ week, and the responses were comparable in both the immunized groups. Chickens immunized with either *Salmonella*-M2e-NA or *Salmonella*-HA-M2e-NA based vaccine elicited significantly (*p* < 0.05) higher NA-specific IgG responses compared to the control group (Figure 2B). The IgG levels were detected at 14^th^ day post-immunization, which peaked at 6^th^ week in both the vaccinated groups (Figure 2B). Our results further demonstrated that the HA and the NA-specific IgG levels were comparable among immunized groups (*p* > 0.05). Chickens immunized with either *Salmonella*-M2e, *Salmonella*-M2e-NA or *Salmonella*-HA-M2e-NA based vaccine elicited significantly (*p* < 0.05) higher M2e-specific IgG responses compared to the control group (Figure 2C). The IgG levels were detected at 14^th^ day post-immunization, which peaked at 28^th^ day and then maintained till 6^th^ week. There were no statistically significantly differences in M2e-specific IgG responses among the immunized groups, however, the M2e-specific responses were significantly (*p* < 0.05) higher at 4 and 6^th^ week post-immunization compared to the HA and the NA-specific IgG responses (Figure 2C).

We also measured mucosal IgA responses in immunized chickens at 28^th^ day post-immunization (Figures 2D–F). Our results showed that chicken vaccinated with *Salmonella*-HA-M2e-NA based vaccine showed significantly (*p* < 0.05) higher HA (Figure 2D), M2e (Figure 2E) and NA (Figure 2F) specific IgA levels compared to the other immunization groups. Further, chickens immunized with either *Salmonella*-M2e or *Salmonella*-M2e-NA based vaccine elicited significantly (*p* < 0.05) higher M2e specific IgA responses compared to the control group (Figure 2F). Although chickens immunized with either *Salmonella*-M2e-NA or *Salmonella*-HA based vaccine showed slight increase in NA and HA-specific IgA mucosal responses, respectively, but the responses were statistically non-significant (*p* > 0.05) compared to the control chicken group.

### Immunization with HA, M2e and NA antigens delivered by *Salmonella* system activates efficient CMI responses

**Figure 3:**
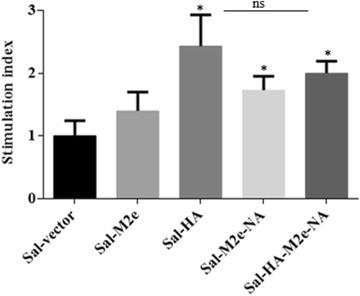
**In vitro proliferations of lymphocytes from vaccinated chickens in response to recall inactivated H7N9 antigen.** Chickens (*N* = 12) were immunized with Sal-vector, Sal-HA, Sal-M2e, Sal-M2e-NA or Sal-HA-M2e-NA vaccine, and PBMCs from vaccinated chickens after 14 days post-immunization were restimulated with inactivated H7N9 antigen (5 µg/mL) for 72 h and lymphocyte proliferation was determined by MTT assay. Results are expressed as stimulation indices, defined as proliferation in response to recall antigen relative to the mock stimulated cells. Each data points represent mean ± SD of five chickens per group.*p < 0.05. ns: non-significant.

To assess the efficacy of influenza-specific CMI responses stimulated by *Salmonella*-based influenza vaccines, the proliferative capacity of vaccinated PBMCs after in vitro stimulation in the presence of inactivated H7N9 recall antigen (5 µg/mL) was evaluated. Efficient proliferative responses were only observed in vaccinated chickens compared to the control chickens (Figure 3). Among immunized groups, recall responses were higher in chickens vaccinated with *Salmonella*-HA, *Salmonella*-M2e-NA or *Salmonella*-HA-M2e-NA based vaccine compared to the chickens immunized with *Salmonella*-M2e based vaccine (Figure 3).

**Figure 4:**
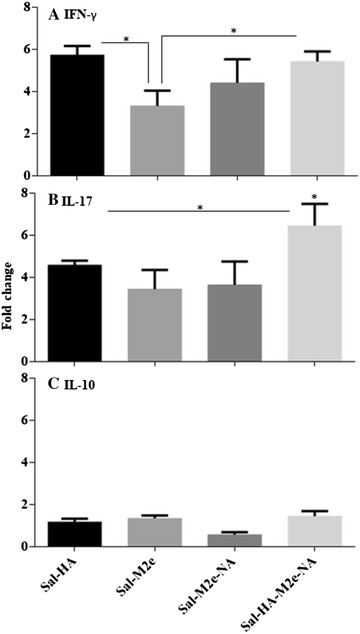
**qRT-PCR analysis of cytokine gene expressions in PBMCs after stimulation with inactivated H7N9 antigen.** Chickens (*N* = 12) were immunized with Sal-vector, Sal-HA, Sal-M2e, Sal-M2e-NA or Sal-HA-M2e-NA vaccine, and PBMCs from immunized chickens after 14 days post-immunization were stimulated with inactivated H7N9 antigen for 24 h and analysed for induction of IFN-ϒ (**A**), IL-17 (**B**) and IL-10 (**C**) mRNA transcription levels by qRT-PCR assay. Results are expressed as fold change in mRNA transcription after inactivated H7N9 antigen stimulation of immunized PBMCs compared to the inactivated H7N9 treated naïve PBMCs. Gene expressions were normalized to GAPDH and mRNA levels of naive stimulated cells were used as calibrator. Data presented are mean ± SD of five chickens per group. *p < 0.05. ns: non-significant.

To complement the study of influenza-specific CMI responses stimulated by *Salmonella*-based influenza vaccines, we evaluated the capacity of vaccinated PBMCs to produce IFN-γ, IL-17 and IL-10 cytokines in response to the stimulation with the inactivated H7N9 recall antigen (5 µg/mL). The mRNA transcript levels of these cytokines were significantly (*p* < 0.05) higher in vaccinated chickens compared to the control chickens (Figure 4). The mRNA inductions of IFN-γ and IL-17 showed 3.3–5.7-folds and 3.6–6.4-folds, respectively, increase in comparison to the stimulated control chicken PBMCs (Figures 4A and B). Among immunized chickens, the IFN-γ levels were significantly (*p* < 0.05) higher in Sal-HA and Sal-HA-M2e-NA groups compared to the Sal-M2e and Sal-M2e-NA groups. With regard to IL-17, the levels were significantly (*p* < 0.05) higher in Sal-HA-M2e-NA group compared to the other immunized groups, which showed almost comparable levels (*p* > 0.05). The IL-10 mRNA levels showed 0.6–1.4-folds increase in comparison to the stimulated naïve PBMCs (Figure 4C). All the immunized groups have shown comparable IL-10 levels; however, the IL-10 responses were significantly (*p* < 0.05) lower compared to the IFN-γ and IL-17 levels, indicating Th1 type of immunity. Our results, thus, indicate that in vitro re-stimulation of vaccinated PBMCs with inactivated H7N9 antigen resulted in efficient recall cellular activation, and *Salmonella*-based influenza antigens have the potential to elicit Th1 type of immunity, which is essential for the clearance of influenza infections [21].

### Immunization with HA, M2e and NA antigens delivered by *Salmonella* system protects chickens against lethal H7N9 challenge

**Figure 5:**
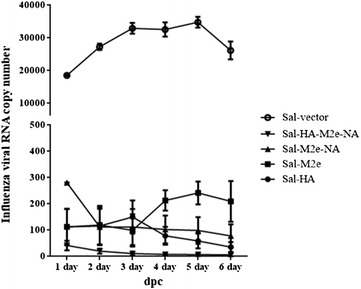
**Protective efficacies of the** ***Salmonella*-based influenza vaccines against LPAI H7N9 challenge.** Chickens (*N* = 12) were immunized with Sal-vector, Sal-HA, Sal-M2e, Sal-M2e-NA or Sal-HA-M2e-NA vaccine, and 28 days later all the immunized chickens were challenged with 1.4 × 10^4^ TCID_50_ H7N9 virus. The protective efficacy was determined by estimation of H7N9 viral RNA copy numbers in the cloacal swab samples of the immunized chickens (*n* = 10) after challenge with the lethal H7N9 virus. dpc: days post-challenge.

Protection against the lethal virus challenge can determine the efficacy of a vaccine. Therefore to evaluate the protective efficacy of a *Salmonella*-based influenza vaccine, all the vaccinated and the control chickens were intranasally challenged with 1.4 × 10^4^ TCID_50_ of H7N9 virus. Subsequently, cloacal wash samples were collected from day 1 to day 6 post-challenge for determination of viral load by qRT-PCR assay and the results were expressed as percent positive for the presence of viral RNA. The presence of viral RNA was found in all the groups from day 1 to 6, albeit, chickens vaccinated with influenza antigens showed significantly (*p* < 0.05) lower viral load compared to the control chicken group (Figure 5). Among immunized groups, chickens which received a co-mix of HA, M2e and NA antigens showed significantly (*p* < 0.05) lower viral load from day 1 to day 6 compared to the other vaccinated groups, indicating that the coadministration of influenza antigens delivered by *Salmonella* system enhanced protective immune responses and *Salmonella*-based influenza vaccines can protect the hosts against lethal influenza challenges. In case of Sal-M2e group, the viral load in immunized birds had not shown a constant trend. On day 2 and 3 post-challenge the viral RNA was lower compared to the viral load measured at other days following challenge with H7N9, suggesting that immunization with Sal-M2e vaccine has considerably delayed the viral replication in vaccinated birds and thus influenza infection. Although the immunized birds had shown viral load in fecal samples until day 6 post-challenge, however, vaccination with *Salmonella*-based influenza vaccines had efficiently contained the H7N9 viral infection and thus considerably reduced the viral shedding in birds (Figure 5).

**Figure 6:**
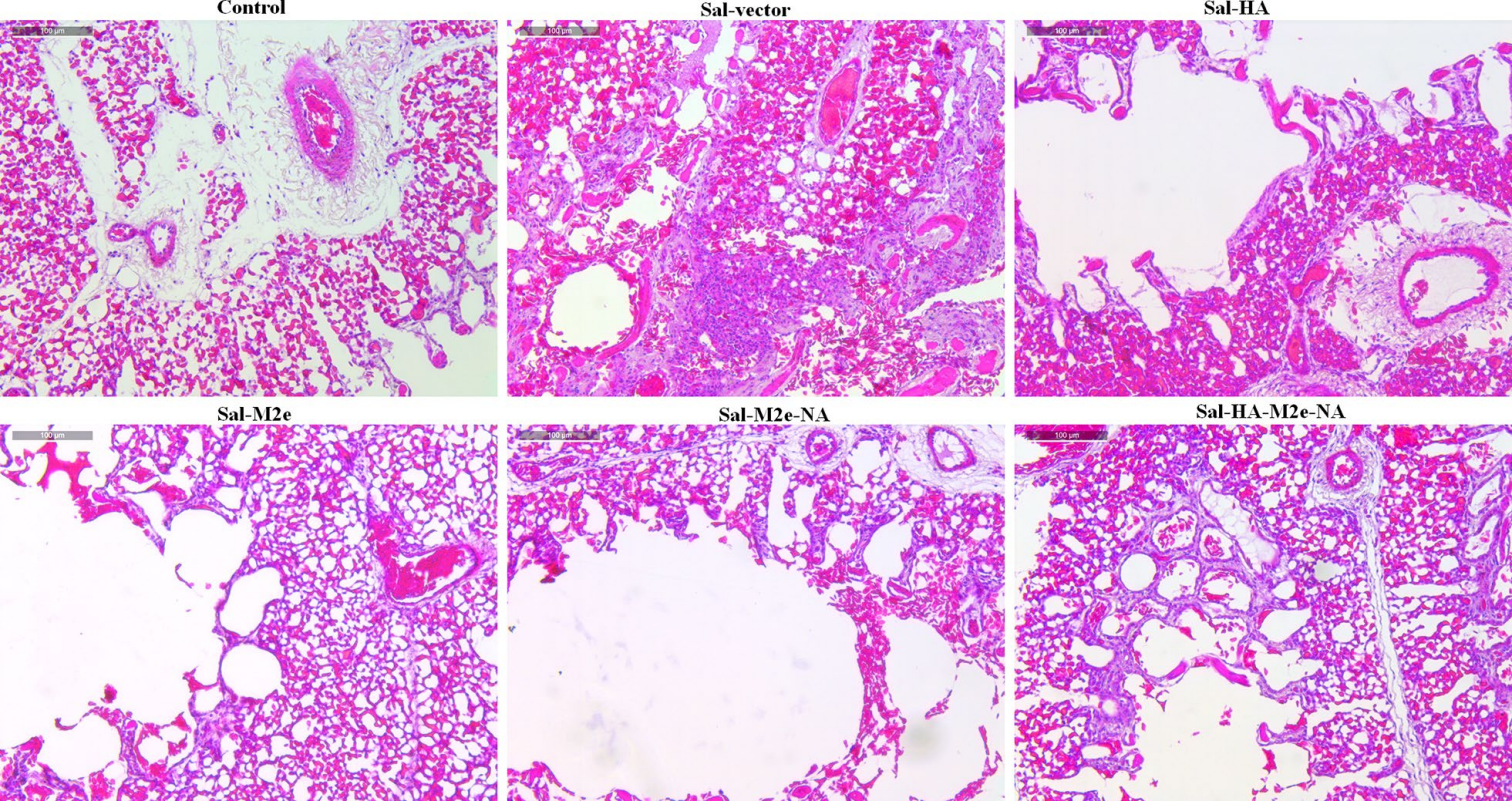
**Photomicrographs of hematoxylin-and eosin-stained lung sections of chickens on 4**^**th**^** day post-challenge. **Chickens (*N* = 12) were immunized with Sal-vector, Sal-HA, Sal-M2e, Sal-M2e-NA or Sal-HA-M2e-NA vaccine, and 28 days later all the immunized chickens were challenged with 1.4 × 10^4^ TCID_50_ H7N9 virus. At 4^th^ day post challenge, chickens (*n* = 3) were sacrificed and lung tissues were collected for histological analysis.

**Figure 7:**
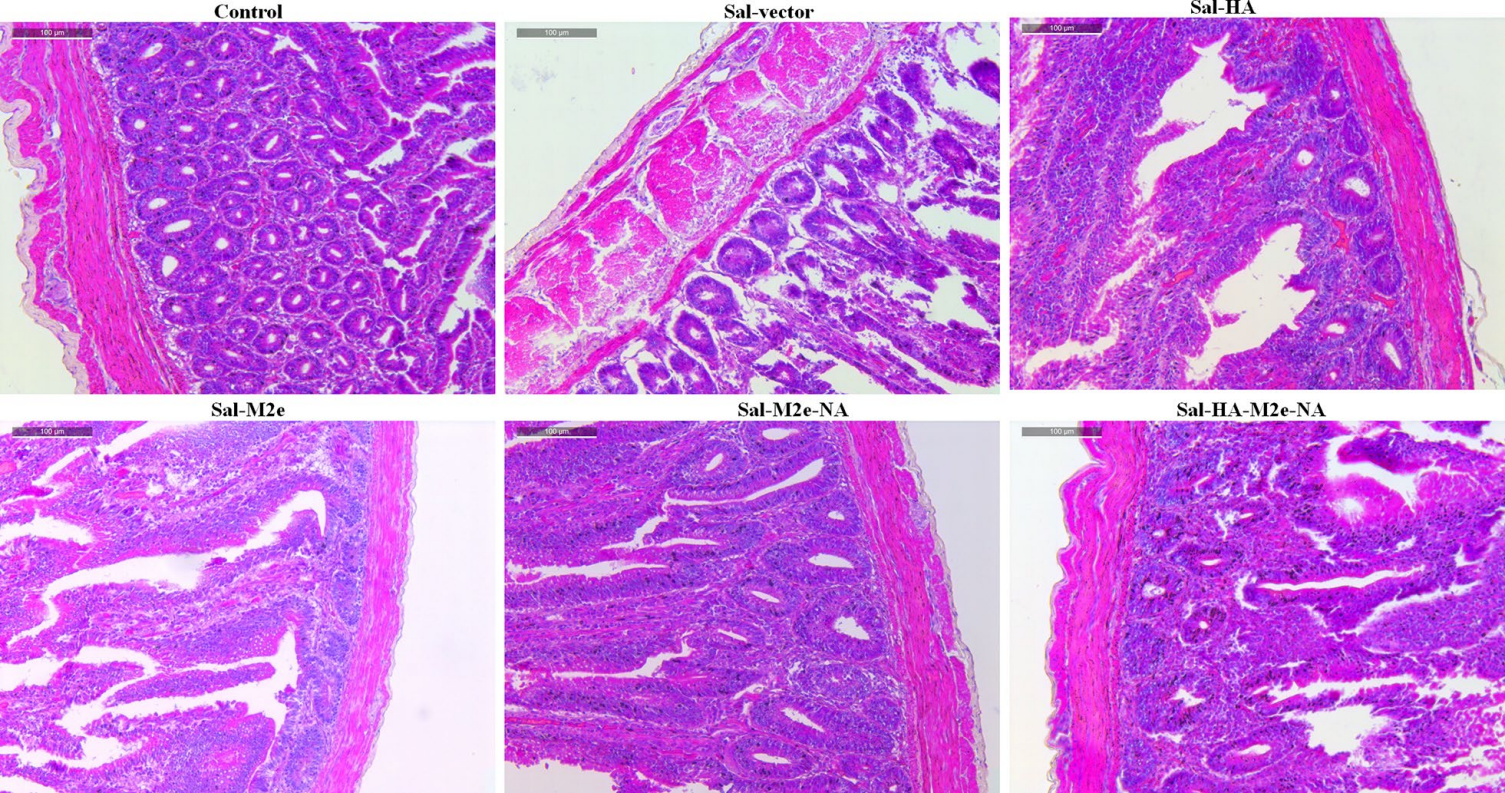
**Photomicrographs of hematoxylin-and eosin-stained intestinal sections of chickens on 4**^**th**^** day post-challenge.** Chickens (*N* = 12) were immunized with Sal-vector, Sal-HA, Sal-M2e, Sal-M2e-NA or Sal-HA-M2e-NA vaccine, and 28 days later all the immunized chickens were challenged with 1.4 × 10^4^ TCID_50_ H7N9 virus. At 4^th^ day post challenge, chickens (*n* = 3) were sacrificed and intestinal tissues were collected for histological analysis.

We further investigated the effect of *Salmonella*-based vaccines on virus-specific immune protection by the histological examination of lung and intestinal tissues of chickens on 4^th^ day post H7N9 challenge. As expected, lung (Figure 6) and intestinal tissues (Figure 7) of uninfected chickens were normal. Chickens treated with *Salmonella* vector had pulmonary (Figure 6) and intestinal lesions (Figure 7), while as chickens vaccinated with Sal-HA, Sal-M2e, Sal-M2e-NA or Sal-HA-M2e-NA vaccines exhibited minimal lesions compared to the Sal-vector group. All these findings suggest that *Salmonella*-based vaccines can offer significant protection against the influenza viruses.

## Discussion

The present study demonstrates the potential of *Salmonella*-based influenza vaccines to elicitate efficient antigen-specific immune responses and protection against a lethal H7N9 infection in chicken model. A major obstacle in influenza vaccine development is the extent of genetic diversity among influenza strains and therefore, neutralizing antibodies elicited by one vaccine strain provides little or no protection against the heterologous strain [5]. In accordance with this notion, we generated HA and NA consensus sequences to centralize the immunogenicity of the vaccine antigen that might provide cross protection against various circulating strains of influenza viruses. Another approach to achieve a broad spectrum of protection is the designing of vaccines based on the highly conserved viral antigens that are subjected to minor antigenic variations. M2e is highly conserved target and an attractive choice for the development of a universal influenza A vaccine [22, 23]. In the present study, we used four tandem repeats of M2e to increase the immunogenicity of M2e antigen [23]. Earlier studies show that M2e-based vaccines induced certain level of cross protective immunity in animal models, but partial protection against the lethal influenza challenges [23, 24]. The coadministration of M2e with either HA or NA is an effective strategy to induce broadly cross-reactive anti-M2e specific antibodies. Previous study shows that immunization with Freund’s adjuvanted M2e antigen failed to induce M2e-specific antibody responses, but by administering HA-M2e conjugate readily elicited M2e specific antibodies [25]. Thus in the present study, we used M2e either alone or in combination with HA and NA proteins. Previously, we show that *Salmonella*-HA consensus based vaccine provided efficient protection against heterologous H7N1 infection [15]. Herein, we report that immunization with *Salmonella* mutants encoding HA, NA and M2e antigens, respectively, administered either alone or in combination, provided significant protection against the lethal H7N9 infection, however, the protective efficacy was higher in *Salmonella*-HA-M2e-NA vaccine compared to the *Salmonella*-M2e or *Salmonella*-M2e-NA based vaccine. *Salmonella*-based vaccination strategy has certain advantages over conventional influenza vaccines. This vaccination strategy is readily and rapidly amenable to modifications once the circulating strains are identified, and *Salmonella*-based vaccines can be rapidly produced in large quantities without the need of specific cell culture conditions required for the production of inactivated whole virus based influenza vaccines. Further, *Salmonella* system acts as a natural adjuvant and delivers efficient maturation signals to dendritic cells, which is essential for the development of effective antigen-specific adaptive immune responses [18, 26, 27].

Elicitation of influenza-specific neutralizing antibody responses following infection/vaccination in peripheral blood circulation strongly correlates with the recovery from the clinical disease and protection from subsequent influenza infection [14]. The present study demonstrates that immunization with *Salmonella*-HA and *Salmonella*-HA-M2e-NA based vaccines elicited higher levels of neutralizing antibody responses compared to the *Salmonella*-M2e-NA and *Salmonella*-M2e immunized groups and this correlates well with the challenge studies. Our immunization study shows that *Salmonella*-HA-M2e-NA based vaccine elicited higher protective immunity compared to the *Salmonella*-HA, *Salmonella*-M2e or *Salmonella*-M2e-NA vaccine, as indicated by the reduced viral shedding in the cloacal samples. Several studies demonstrated that mucosal immunization could result in the induction of higher immune responses not only at the site of stimulation but also in remote external secretions, and mucosal IgA antibodies were more effective and cross-protective against viral infection than systemic IgG antibodies [28, 29]. The induction of antigen-specific secretory IgA antibodies following infection/vaccination at mucosal surfaces prevent viral attachment to epithelial cells and infection of the host cells subsequently [30, 31]. In the present study, we found that chickens vaccinated with *Salmonella*-HA-M2e-NA based vaccine elicited higher mucosal HA, M2e and NA-specific IgA responses compared to the chickens immunized with *Salmonella*-HA, *Salmonella*-M2e or *Salmonella*-M2e-NA based vaccine. This might explain why *Salmoenlla*-HA-M2e-NA vaccinated chickens show higher protection rate against the lethal H7N7 virus infection. Thus, our data supports the conclusion that orally administered *Salmonella*-based vaccines can enhance systemic as well as mucosal immune responses, and the co-administration of HA, M2e and NA antigens improves mucosal and protective immune responses in chickens. Studies have shown that M2e specific antibodies mediate cross protection against the influenza A virus infections [23]. Thus, our vaccination strategy could provide broad spectrum of protection against various influenza subtypes and therefore warrants further research in this regard.

The induction of cellular immune responses following influenza infection are necessary for viral clearance from the lungs and are, therefore, critical for chickens to recover from the clinical disease [21]. In the present study, the *Salmonella*-based HA, NA and M2e vaccines not only generated efficient humoral responses but also stimulated high level of antigen-specific cellular responses. Previous study have shown that immunization with *Salmonella*-based HA and NA vaccination elicits efficient cellular immune responses and protects the mice against the homologous and heterologous H5N1 and H1N1 challenges, respectively [32]. Our results show that vaccinated PBMCs stimulated with recall inactivated H7N9 antigen resulted in higher proliferative responses compared to the control chickens. Further, we evaluated the recall cytokine responses in vaccinated PBMCs. The nature of the cytokines secreted after vaccination or in vitro restimulation of cells is an important parameter to define the type of immunity stimulated. Our data demonstrates that chickens immunized with *Salmonella*-HA-M2e-NA based vaccine showed higher IFN-γ, a Th1 cytokine, and IL-17, a proinflammatory cytokine, recall responses compared to the *Salmonella*-HA, *Salmonella*-M2e or *Salmonella*-M2e-NA vaccinated chickens. IFN-γ is critical in the development of CMI responses, especially cytotoxic CD8^+^ responses, which are more potent and effective in clearance of viral infections [33, 34]. This might explain why *Salmonella*-HA-M2e-NA immunized chickens cleared H7N9 infection at a faster rate than the other immunized groups. The IL-17 plays important and protective roles at host epithelial and mucosal barriers against certain pathogens [35]. A report by Wang et al. shows that IL-17 deficient mice exhibit markedly increased weight loss, more pronounced lung immunopathology, and significantly reduced survival rates upon infection with lethal H5N1 virus [36]. Another report has shown that IL-17 can protect mice against lethal H1N1 (A/Puerto Rico/8/34) and H3N2 (A/Alaska/6/77) virus challenges [37]. This might further explain why *Salmonella*-HA-M2e-NA vaccinated chickens showed enhanced protection against the lethal H7N7 influenza virus challenge. We also observed an increment in IL-10, the Th2 cytokine; however, the IL-10 levels were significantly lower compared to IL-17 and IFN-γ, indicating elicitation of Th1 type of immunity, which is important for the clearance of viral infections. These findings clearly indicate that *Salmonella*-based influenza vaccines can stimulate efficient Th1 type of immunity and can protect the hosts from pathogenic influenza infections. Our results are in agreement to the previously published reports [18, 32].

In summary, we show that *Salmonella*-based influenza vaccines elicitate efficient induction of antigen-specific humoral and CMI responses, and offered significant protection against the LPAI H7N9 infection in chickens. Thus, *Salmonella*-based vaccination strategy could be used as a potential prophylactic approach against LPAI viruses, which generally emerge to HPAI viruses affecting both animal and human population. Further, this approach can be readily and rapidly adjusted to the changes in the circulating field strains of influenza virus and, therefore, this promising technology can overcome the limitations usually associated with the conventional inactivated influenza vaccines, including SPF embryonated eggs and a long time line that could be threatened during an influenza pandemic.

## Additional files

**Additional file 1.** **Phylogenetic analysis of the H7N9 NA sequences.** The Clustal W algorithm was used to generate the NA consensus sequence of H7N9 virus based on the data available in the Influenza Virus Resource data base in NCBI.

**Additional file 2.** **Western blot analysis of NA protein expressed by JOL2052.** The expression of NA protein was confirmed by Western blot analysis. The JOL2052 bacteria harbouring pMMP65-NA plasmid and JOL1837 bacteria (serving as control) were allowed to grow till 0.6 OD_600nm_. Then bacterial pellets were subjected to Western blot analysis using polyclonal NA antibody (catalog#, NBP2-41279, Novus Biologicals USA). Lane M, Protein Marker (catalog#, P8500, GenDEPOT, USA); lane 1, control bacterial pellet, and lane 2, bacterial pellet of JOL2052 showing a 58 kDa band.
