# Supplementary figures and images for: Oral immunization with a novel attenuated Salmonella Typhimurium encoding influenza HA, M2e and NA antigens protects chickens against H7N9 infection
Source: Vet Res. 2018 Feb 1;49:12. doi: 10.1186/s13567-018-0509-y (PMC5796500; doi:10.1186/s13567-018-0509-y)

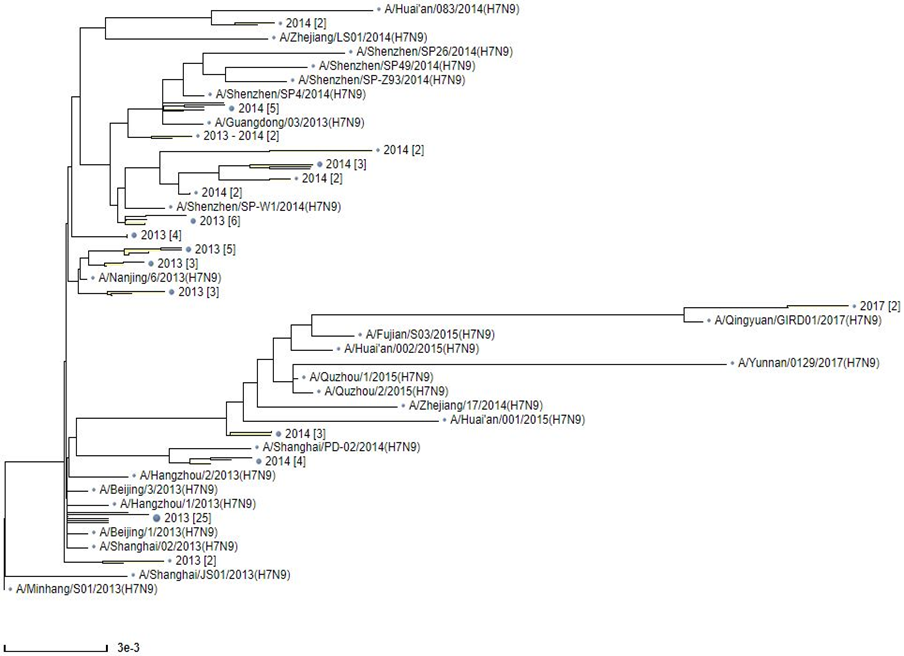

Supplement: Supplementary file 1 — Additional file 1. Phylogenetic analysis of the H7N9 NA sequences. The Clustal W algorithm was used to generate the NA consensus sequence of H7N9 virus based on the data available in the Influenza Virus Resource data base in NCBI. [file 13567_2018_509_MOESM1_ESM.tif]

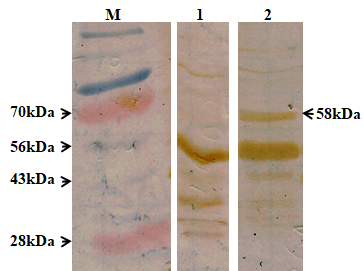

Supplement: Supplementary file 2 — Additional file 2. Western blot analysis of NA protein expressed by JOL2052. The expression of NA protein was confirmed by Western blot analysis. The JOL2052 bacteria harbouring pMMP65-NA plasmid and JOL1837 bacteria (serving as control) were allowed to grow till 0.6 OD600nm. Then bacterial pellets were subjected to Western blot analysis using polyclonal NA antibody (catalog#, NBP2-41279, Novus Biologicals USA). Lane M, Protein Marker (catalog#, P8500, GenDEPOT, USA); lane 1, control bacterial pellet, and lane 2, bacterial pellet of JOL2052 showing a 58 kDa band. [file 13567_2018_509_MOESM2_ESM.tif]
